# A randomised controlled study of dog assisted therapy: comparing a short and a long version for patients with fetal alcohol spectrum disorder

**DOI:** 10.3389/fpsyg.2026.1720834

**Published:** 2026-04-07

**Authors:** Silvia Muñoz-Caller, Raquel Vidal, Jorge Lugo, Francesc Ristol, Anna Veiga, Cristina Vico, Josep Antoni Ramos-Quiroga, Nuria Gómez-Barros

**Affiliations:** 1 Department of Psychiatry, Hospital Universitari Vall d’Hebron, Barcelona, Catalonia, Spain; 2 Group of Psychiatry, Mental Health and Addiction, Vall d’Hebron Research Institute (VHIR), Barcelona, Spain; 3 Department of Psychiatry and Legal Medicine, Universitat Autònoma de Barcelona, Barcelona, Spain; 4 Biomedical Network Research Centre on Mental Health (CIBERSAM), Instituto de Salud Carlos III, Madrid, Spain; 5 Centre of Dog Assisted Therapy (CTAC), Barcelona, Spain; 6 Fundació Probitas, Barcelona, Spain

**Keywords:** animal assisted therapy (AAT), caregiver depression and anxiety, children and adolescents interventions, dog assisted therapy (DAT), fetal alcohol syndrome disorder, treatment duration

## Abstract

**Background:**

Fetal Alcohol Spectrum Disorder (FASD) presents with complex neurodevelopmental challenges, including emotional, behavioral, and social difficulties. Dog-Assisted Therapy (DAT) has shown potential benefits, but optimal duration for maximal efficacy remains unclear.

**Objective:**

The present randomised controlled trial aimed to compare the therapeutic impact of a short version (8 weekly sessions) versus a long version (16 sessions) of a manualised DAT protocol on clinical, functional, and family outcomes in children and adolescents with FASD.

**Methods:**

Sixty-four participants were randomised to the short or long DAT version, with 55 completing treatment. Primary outcomes included social skills, internalizing and externalizing symptom profiles, quality of life, clinical severity, and parental anxiety and depression.

**Results:**

Both groups showed significant improvements across externalizing symptoms, social skills, FASD severity, and quality of life. However, the long-version DAT group exhibited greater reductions in externalizing symptoms (CBCL Inattention: *F*(1, 54) = 4.68, *p* = 0.035, ηp^2^ = 0.083), problem behaviors (SSIS-P: *F*(1, 54) = 7.80, *p* = 0.007, ηp^2^ = 0.13), and lower clinician- and parent-rated FASD severity scores (CGI-S Clinician: *F*(1, 54) = 6.54, *p* = 0.014, ηp^2^ = 0.112; CGI-S Parent: *F*(1, 54) = 4.94, *p* = 0.031, ηp^2^ = 0.087). Enhanced quality of life was also observed (KIDSCREEN-27 Peers and Social Support: *F*(1, 54) = 4.39, *p* = 0.041, ηp^2^ = 0.078). Additionally, caregivers in the long-version group reported significant reductions in depressive symptoms (BDI-II: *F*(1, 54) = 14.03, *p* < 0.001, ηp^2^ = 0.212). Both versions improved anxiety metrics comparably.

**Clinical trial registration:**

clinicaltrials.gov, NCT06763614.

## 1 Introduction

Fetal Alcohol Spectrum Disorder (FASD) is a complex neurodevelopmental syndrome caused by prenatal alcohol exposure, resulting in a broad spectrum of cognitive, emotional, physical, and behavioral challenges. It is estimated to affect between 1 and 5% of the global population, although prevalence varies by region and diagnostic criteria (May et al., 2018; Popova et al., 2023). FASD is frequently accompanied by co-occurring neurodevelopmental conditions, including Attention Deficit Hyperactivity Disorder (ADHD) and Intellectual and Developmental Disorders (IDD) (Attell et al., 2025). The overlap of these conditions complicates diagnosis and management and contributes to the heterogeneity of FASD clinical presentations.

Individuals with FASD often experience persistent challenges throughout their lives, including intellectual disabilities, altered social skills, difficulties regulating emotions, and behavioral challenges such as impulsivity, oppositionality, and inattention. These concerns significantly impact academic achievement, interpersonal relationships, and adaptive functioning. Additionally, the caregiving demands associated with managing these behavioral and cognitive needs place significant emotional and psychological burdens on caregivers and families, reducing their quality of life and increasing stress (Mukherjee et al., 2013).

According to Hoyme et al. (2016), FASD comprises three subtypes. Fetal Alcohol Syndrome (FAS) requires: (May et al., 2018) specific facial dysmorphology, Popova et al. (2023) prenatal/postnatal growth <10th percentile, Attell et al. (2025) neurologic features (e.g., small head circumference, structural brain abnormalities, or recurrent nonfebrile seizures), and (Mukherjee et al., 2013) a specific pattern of neurobehavioral challenges -without needing confirmed prenatal alcohol exposure (PAE). Partial FAS (pFAS) includes facial features plus neurobehavioral challenges but lacks growth/neurologic criteria. ARND requires confirmed PAE plus neurobehavioral challenges without facial features.

Psychological interventions aimed at improving emotional regulation and social skills have demonstrated promise in enhancing functioning among those with FASD (Flake et al., 2025; Paley and O’Connor, 2011). Such programs are associated with reductions in externalizing behaviors and improvements in social competence (Keil et al., 2010). Despite these advances, there remains a need for innovative, engaging, and tailored therapeutic approaches suited to the unique needs of this population.

Animal-Assisted Therapy (AAT), particularly Dog-Assisted Therapy (DAT), has gained increasing attention as an effective adjunct for neurodevelopmental disorders (Gómez-Calcerrada et al., 2021; Carlisle et al., 2025; Schuck et al., 2018). DAT harnesses the human-animal bond to foster emotional regulation, social interaction, and overall well-being (Narvekar and Krishnan, 2024; Narvekar and Narvekar, 2022). Therapy dogs provide not only companionship but also sensory regulation and social facilitation, collectively creating a safe, supportive therapeutic environment conducive to skill acquisition. These mechanisms are especially pertinent in FASD, where individuals often demonstrate unique relational strengths, sensory processing capacities, and social intuition that therapy dogs can leverage to support attention, emotional regulation, and social reciprocity challenges -common concerns often resistant to conventional interventions. (Hüsgen et al., 2022; Guillen Guzmán et al., 2022; Vincent et al., 2014). This strengths-based approach capitalizes on FASD-specific assets while addressing functional needs.

Although prior studies demonstrate the efficacy of DAT for neurodevelopmental disorders, including FASD (Vidal et al., 2023; Vidal et al., 2020), the optimal treatment duration has not yet been empirically established, as existing protocols range widely from approximately 6 to 20 sessions and lack direct comparisons of treatment “dose.” Evidence from dose–response models in behavioral interventions suggests that longer treatment versions may yield greater improvements in domains such as attention and social functioning; however, this proposition has not yet been directly tested within DAT frameworks. Establishing the most effective DAT “dose” is therefore critical for the development of cost-effective and sustainable intervention protocols. Furthermore, because FASD-related challenges are closely associated with elevated parental anxiety and depressive symptoms (Harding et al., 2022; Ilchena et al., 2023; Lach et al., 2009), evaluation of caregiver outcomes is warranted. Although child-focused interventions have been shown to confer secondary benefits for caregivers (Purpura et al., 2021), this relationship has not been systematically examined in the context of DAT.

**Figure 1:**
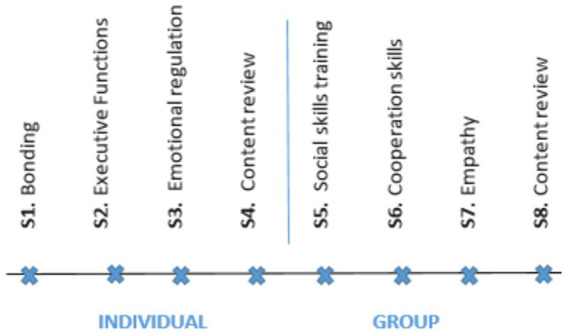
Sessions flow and contents for the short version.

**Figure 2:**
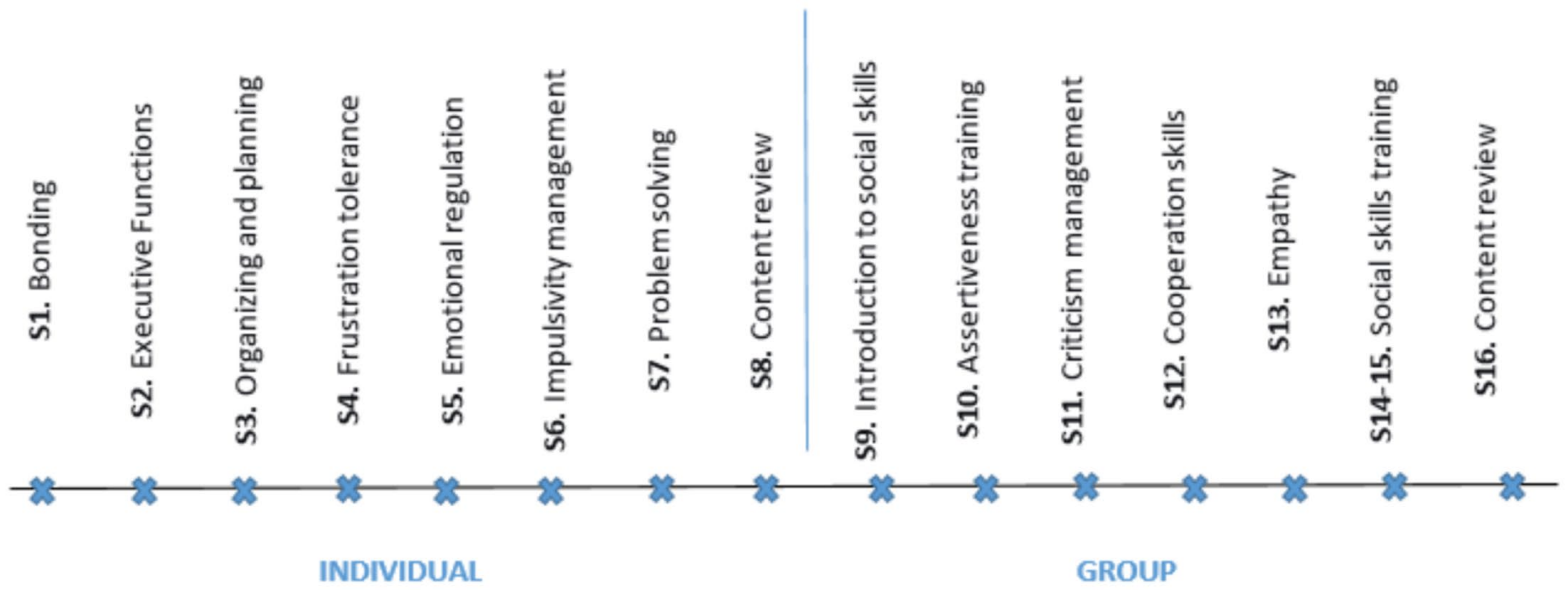
Sessions flow and contents for the long version.

This manualized DAT protocol positions therapy dogs as active co-therapists targeting FASD core needs. Dogs facilitate executive function (turn-taking with commands), model emotional regulation (frustration tolerance tasks), guide problem-solving (interactive play), and structure social skills practice (group dog-handling scenarios), with canine activities explicitly mapped to session objectives (Figures 1, 2).

Evidence from dose–response models in behavioral interventions for neurodevelopmental disorders suggests longer exposures yield superior gains in attention, executive function, and social skills (Corrales et al., 2024; Linstead et al., 2017). In DAT specifically, existing protocols range 6–20 sessions without comparative testing, despite cumulative canine exposure hypothesized to enhance autonomic regulation and skill generalization (Hüsgen et al., 2022).

Long DAT versions may allow for more sustained and cumulative engagement with these mechanisms, promoting deeper learning and generalization across settings (Risberg and Edner, 2025; Song et al., 2025). Prolonged exposure to therapy dogs’ calming presence can better support autonomic regulation and stress reduction -foundational for cognitive/emotional control (Yotanyamaneewong et al., 2025). Furthermore, long versions enable comprehensive practice of social interactions and problem-solving in individual and group contexts, fostering heightened social competence and resilience. In contrast, shorter interventions provide rapid behavioral gains but may lack dosage for complex skill consolidation (Mittly et al., 2024). By explicitly examining differential therapy duration impacts, this study tests how these DAT mechanisms translate into clinical gains over time, informing optimized protocols tailored to FASD’s complex needs. Importantly, potential benefits of longer DAT versions may extend beyond the child to caregivers. Given behavioral dysregulation and attentional difficulties in FASD are linked to elevated caregiver stress, anxiety, and depressive symptoms (Purpura et al., 2021; Tait et al., 2025; Mohamed et al., 2020; Paley et al., 2005), child self-regulation/social functioning improvements may indirectly alleviate caregiver burden. Longer interventions thus support deeper skill consolidation in children (particularly attention/social reciprocity) and secondary caregiver wellbeing through reduced behavioral management demands.

Based on these considerations, we hypothesized that both short and long DAT would yield measurable benefits, with the long version producing superior outcomes in domains requiring sustained practice (i.e., attention, social reciprocity). Secondarily, we predicted greater caregiver psychological benefits (depression, anxiety) in the long-DAT version, consistent with secondary parental gains following child-focused interventions in related populations.

FASD affects 1–5% of children globally, imposing lifelong adaptive/academic difficulties and high caregiver morbidity/service costs (Popova et al., 2023; Flannigan et al., 2022; May et al., 2014). With scarce scalable interventions, these findings will inform evidence-based service planning by establishing minimum effective DAT duration, thereby optimizing therapeutic resource allocation for children, adolescents with FASD, and their families.

## 2 Materials and methods

### 2.1 Study design

This study employed a randomised, rater-blinded, controlled trial design. Participants were randomly assigned either to the long version group of 16 sessions (*n* = 33) or to the short version group of 8 sessions of DAT treatment (*n* = 31). A computer program was used to create a random sequence of numbers, which determined the order in which participants were assigned to either the short or long DAT treatment groups.

Block randomization ensured balanced allocation based on key demographic variables, such as age, to minimize potential confounding factors. To prevent selection bias, allocation concealment was implemented, ensuring that those enroling participants were unaware of group assignments until allocation. Raters assessing outcomes were blinded to the participants’ group assignments.

### 2.2 Participants

Patients were recruited from the FASD program at Vall d’Hebron University Hospital in April 2025. A pre-recruitment power analysis indicated that a medium effect size (ηp^2^ ≈ 0.06–0.14) could be detected, consistent with prior behavioral intervention studies for neurodevelopmental disorders. Of the 64 participants randomly assigned, 55 completed the study (short version *n* = 24; long version *n* = 31), reflecting practical enrolment constraints. This sample size afforded adequate statistical power (1–*β* = 0.80, *α* = 0.05) to detect medium-to-large effects.

Inclusion criteria comprised a confirmed FASD diagnosis, age 6–18 years, and stable medication use for at least 2 months prior to enrolment. Participants agreed not to initiate additional psychiatric or psychological treatments during the study. Individuals with borderline intellectual functioning or intellectual disabilities were included to reflect the clinical FASD population.

Exclusion criteria focused specifically on safety considerations, including: behavioral instability or aggression that could pose risks to therapy dogs or other participants; need for intensive treatment (e.g., hospitalization); fear of dogs; allergies to animals; medical or psychiatric conditions that could be exacerbated by canine presence; and any history of inappropriate behavior toward animals.

### 2.3 Treatment content

The study compared two DAT versions: a short version comprising 8 manualised sessions and a long version comprising 16 sessions. Both versions followed the Centre of Dog Assisted Therapy (CTAC) protocol. CTAC is a full-member of the International Association of Human-Animal Interaction Organizations (IAHAIO), a global association of organizations engaged in practice, research, and education in dog-assisted therapy. The CTAC protocol has demonstrated efficacy in prior FASD trials (Vidal et al., 2023; Vidal et al., 2020) and ASD populations (Santaniello et al., 2021), with established fidelity monitoring ensuring replicability.

All interventions were structured into two sequential phases: individual intervention followed by group intervention, administered weekly in 45-min sessions. Each session involved two certified therapy dogs (from 5 total: 3 medium Golden Retriever/Labrador ~30 kg, 2 small Cavalier ~12 kg; ages 2–5), one DAT specialist and a psychologist who facilitated both individual and group modules per session. Patients also received periodic visits from their psychiatrist to monitor medication adherence.

The short version covered the same core content as the long version, but in a condensed form. Its individual phase included four sessions focusing on: building a patient-animal bond (S1), executive and organizational skills (S2), emotional regulation and frustration/anger tolerance (S3), and impulsivity and problem-solving (S4). The subsequent group phase consisted of four sessions addressing: assertive communication and criticism management (S5), cooperation skills and empathy (S6), social skills training (S7), and content review (S8).

The long version maintained this two-phase structure while extending the intervention through additional sessions that provided deeper content exploration and increased opportunities for skill practice and consolidation.

In both versions, the individual phase targeted foundational cognitive skills frequently affected in FASD, including organization, planning, attention regulation, emotional control, impulsivity management, and problem-solving. These were addressed through manualised, evidence-based activities within a supportive environment involving therapy dogs.

The group phase aimed to generalize and practice social skills such as assertiveness, cooperation, empathy, and managing criticism. Group sessions leveraged the social facilitation effect of therapy dogs to enhance engagement and promote positive peer interactions.

The continuous presence of therapy dogs aimed to optimize motivation, reduce anxiety, and support sensory integration, tailoring the approach to the neurodevelopmental profile of children with FASD.

### 2.4 Diagnostic and outcome measures

An initial evaluation was conducted by a team of experts from various fields, including a geneticist specializing in dysmorphology assessment, a neuropsychologist for cognitive testing, and a psychiatrist for evaluating behavior and any co-occurring conditions.

The study employed a comprehensive set of validated measures to capture multiple relevant domains in children with FASD and their caregivers. Outcome measures were administered at pre-treatment (baseline) and at the end of the treatment.

- The Child Behavior Checklist (CBCL) (Achenbach and Edelbrock, 1991) is a widely used, standardized parent-report instrument assessing a broad range of emotional and behavioral problems in young people. The 113-item scale covers internalizing symptoms (e.g., withdrawal, anxiety/depression, somatic complaints), attention problems, thought disturbances, and externalizing behaviors such as oppositionality and conduct issues. Given the high prevalence of such concerns in FASD, the CBCL provides reliable and ecologically valid data on symptom changes. It has been validated in Spanish populations with strong psychometric properties (Estrada et al., 2010) and demonstrated excellent internal consistency in the present sample.
- Social Skills Improvement System–Parent Form SSIS-P (Gresham and Elliott, 2008): evaluates social competence alongside problematic behaviors, including those related to hyperactivity/inattention and autism spectrum traits. It aligns well with the study’s aim of assessing social functioning changes, which are core challenges associated with FASD. SSIS-P’s dual focus on strengths and weaknesses supports a nuanced understanding of treatment effects on social skill acquisition and reduction of problematic behavior.
- KIDSCREEN-27 (Ravens-Sieberer et al., 2007): measures health-related quality of life across physical, psychological, autonomy, family, and social domains. Quality of life is frequently affected in people living with FASD due to functional, emotional, and social limitations. KIDSCREEN-27’s multidimensional approach offers valuable insight into broader wellbeing improvements beyond symptom reduction. The instrument is validated across European pediatric populations, supporting cross-cultural applicability.
- The Clinical Global Impression–Severity and Improvement CGI-S/ CGI-I (Rapoport et al., 1985) is a single-item, 7-point clinician-rated scale assessing overall illness severity at the time of evaluation. Clinicians rate patients relative to their experience with similar FASD cases (1 = normal, not at all ill; 7 = among the most extremely ill), considering symptom frequency, intensity, functional impact across domains like behavior, social skills, emotional regulation, attention, and comorbidities (e.g., ADHD, learning and cognitive difficulties). This complements parent reports to reduce bias and enhance objectivity.
- The Parent Global Impression–Severity and Improvement PGI-S/I: Parents rated their child’s overall severity and improvement using a parent-adapted global impression scale, 7-point scales analogous to the clinician CGI-S and CGI-I. These parent ratings have been previously used in pediatric clinical trials (Wehmeier et al., 2008) and correlate with clinician global impressions.

All measures (except for CGI-S completed by the clinician) were completed by caregivers as patients with FASD often exhibit limited altered awareness, and the reliability of their self-reports may be uncertain. Together, these instruments form a robust measurement battery addressing the multi-faceted impacts of DAT on children with FASD and their caregivers’ emotional health.

To assess emotional symptoms in caregivers, two standardized and validated self-report instruments were administered:

- State–Trait Anxiety Inventory STAI (Spielberger et al., 1971): This tool measures both transient or situational anxiety (state) and relatively stable anxiety proneness (trait), capturing different dimensions of caregiver anxiety that are particularly relevant given the chronic stresses associated with caring for children with FASD.
- Beck Depression Inventory-II BDI-II (Beck et al., 1996): A widely used measure of depressive symptom severity, to quantify caregiver mood disturbances, which commonly co-occur in these populations and may be influenced by effective child-centered interventions.

The study also collected key participant and clinical variables to characterize the sample and support comprehensive analyses:

- Sociodemographic characteristics: Including age and gender to describe basic sample demographics.
- FASD subtype (Hoyme et al., 2016): FAS, pFAS and ARND–to characterize phenotypic heterogeneity and examine potential subtype-specific treatment responses.
- Pharmacological treatment status: Documenting medication usage and adherence to account for potential confounding effects.
- Psychiatric comorbidity was assessed using structured diagnostic interviews: Structured Clinical Interview for DSM Disorders (SCID-I/II) for older adolescents aged 16–18 (*n* = 37 across groups) and Kiddie Schedule for Affective Disorders and Schizophrenia (K-SADS) for participants under 16 years (*n* = 18 across groups). SCID-I/II and K-SADS comprehensively assessed all DSM-5 psychiatric disorders (Table 1 reports primary conditions; full profile available upon request). These assessments, conducted by our multidisciplinary FASD team, characterized baseline co-occurring conditions (Table 1) including ADHD (91–93% prevalence) and learning disorders (16–29%). ASD prevalence was also reported separately (Table 1: 12.5–19.4%).
- Cognitive functioning: Measured via the Wechsler Intelligence Scale for Children or Adults (Wechsler, 1981) to establish intellectual capacity, an important factor influencing treatment engagement and outcomes.

**Table 1:** Participants’ characteristics.

	Short version (*n* = 24)		Long version (*n* = 31)			
Variables	*n*	%	*N*	%	*p*	*X* ^2^
Gender						
Male	14	58.3	16	51.6	0.732	0.117
Comorbidity						
ADHD	22	91.67	29	93.5	0.790	0.071
ASD	3	12.5	6	19.35	0.490	0.460
Learning disorder	7	29.17	5	16.13	0.250	1.350
Diagnosis						
pFAS	11	45.83	9	29.0	0.200	1.650
FAS	9	37.5	19	61.3	0.080	3.063
ARND	4	16.67	3	9.7	0.440	0.595

	Mean	SD	Mean	SD	*p*	*t*
Age	14.33	1.94	13.06	2.75	0.06	1.91
IQ	75.25	14.83	72.16	11.076	0.380	0.885

ADHD, Attention-Deficit/Hyperactivity Disorder; ASD, Autism Spectrum Disorder; FAS, Fetal Alcohol Syndromer; ARND, Alcohol-Related Neurodevelopmental Disorder; IQ, Intellectual Quotient.

These variables serve as essential descriptors of the study population and enable consideration of individual differences in analyses to better understand the effects and generalizability of the DAT interventions.

### 2.5 Procedure

The study was approved by the Ethics Committee of Clinical Investigation of the Vall d’Hebron University Hospital (approval code: PR(AG)055/2025). The DAT intervention was implemented between April and July 2025. During this period of 72 patients approached through the FASD unit, 64 consented to participate after receiving detailed information about the study, three declined to participate and five did not meet inclusion criteria. Of the 64 participant’s enrolled, 55 completed treatment.

Written informed consent was obtained from caregivers, and informed assent was obtained from participants. Following the pre-treatment assessment, the study’s data manager utilized a computerized random number generator (SPSS version 20) to randomly assign participants to the two treatment variations. The raters who conducted the pre-treatment and post-treatment assessments were blinded to the participants’ group assignments (short-version or long-version DAT). These raters were not involved in the delivery of the therapy and had no contact with the research team regarding treatment allocation during the trial.

Participants in the two groups were evaluated at the beginning of the study (baseline) and at the end of the treatment (post). Pre-test assessment was administered 1 week before the beginning of the intervention and the post-test assessment 1 week after the intervention that lasted either 8 or 16 sessions.

### 2.6 Statistical analysis

Data were analyzed (using SPSS version 20). Data were analyzed using SPSS version 20. Analyses included only participants with both pre- and post-treatment assessments (*n* = 55 completers; 86% completion rate). To evaluate treatment effects over time and between groups, each outcome variable was analyzed using a mixed-design repeated-measures ANOVA, with time (pre/post-treatment) as the within-subject factor and treatment group (short vs. long DAT version) as the between-subject factor.

In cases of significant time × group interactions (*p* < 0.05), follow-up simple effects analyses were conducted: paired-samples *t*-tests assessed within-group pre-post changes and independent-samples *t*-tests on change scores (post-pre) compared between-group differences (df = 54). All post-hoc tests were two-tailed (*p* < 0.05) with no multiplicity adjustment due to the limited number of planned primary contrasts. Effect sizes were reported as partial eta squared (ηp^2^) for ANOVAs. Statistical significance was defined as *p* < 0.05.

Normality of residuals and change scores was assessed using Shapiro–Wilk tests for each outcome variable and group. No significant deviations from normality were found (all *p* > 0.05); thus, parametric repeated-measures ANOVA was deemed appropriate. Mauchly’s test of sphericity was non-significant for the within-subject factor (time; *χ*^2^(0) = 0.45, *p* = 0.50), confirming the sphericity assumption; no Greenhouse–Geisser corrections were required.

## 3 Results

### 3.1 Program completion rate

**Figure 3:**
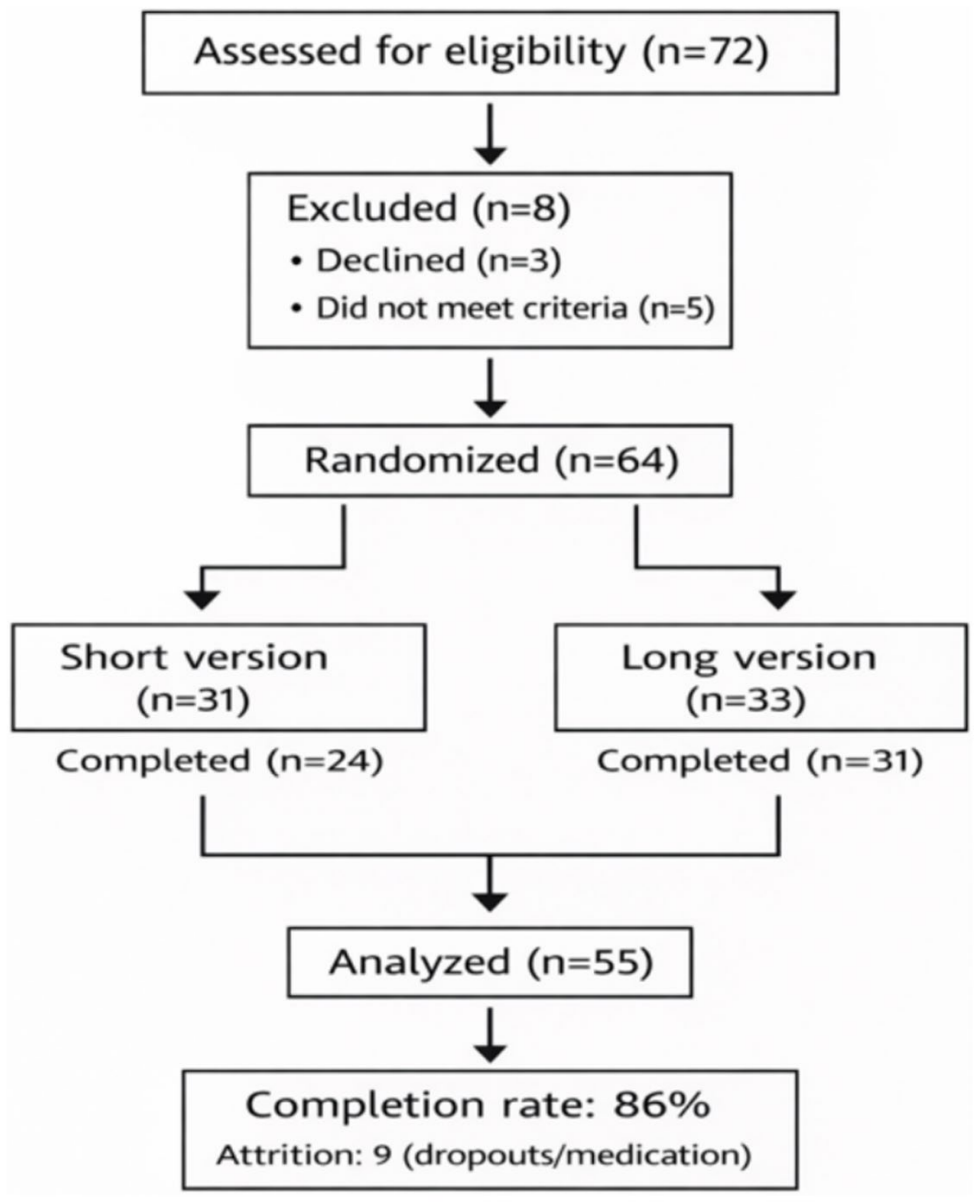
Flow diagram through the phases of the study.

Of the 64 participants initially randomised (short version: *n* = 31; long version: *n* = 33), 55 completed the treatment and were included in the final analysis (short version: *n* = 24; long version: *n* = 31), representing an overall completion rate of 86%. Attrition was due to dropout and medication discontinuation as detailed in Figure 3.

### 3.2 Sample characteristics

The demographic and clinical characteristics of participants across the two treatment groups are summarized in Table 1. No statistically significant differences between groups were detected for any demographic or clinical variables at baseline, confirming successful randomization.

The FASD subtype distribution did not differ significantly between groups (*χ*^2^ = 3.79, *p* = 0.152). The short version group included 37.5% with complete FAS, 45.8% with pFAS, and 16.7% with ARND, while the long version group comprised 61.3% with complete FAS, 29.0% with pFAS, and 9.7% with ARND (see Table 1).

Comorbidity profiles were similar between groups, with ADHD being the most prevalent condition (short: 91.7%; long: 93.5%). Autism spectrum disorder was present in 12.5% of the short version group and 19.4% of the long version group, while learning disorders affected 29.2 and 16.1%, respectively. None of these differences reached statistical significance. Cognitive functioning, as measured by Full Scale IQ, showed comparable scores between groups (short: 85.21 ± 12.34; long: 82.76 ± 14.89; t = 0.648, *p* = 0.521).

### 3.3 Outcome measures results

Significant improvements were observed from pre- to post-treatment across both versions on most outcome measures. Repeated measures ANOVA revealed significant time × group interactions favoring the long version in several key domains, as summarized below and detailed in Table 2.

**Table 2:** Outcome results.

Measures	Short version (*n* = 24)		Long version (*n* = 31)		Time		Time × Group	
	Baseline	Outcome	Baseline	Outcome				
	M (SD)	M (SD)	M (SD)	M (SD)	*F(p)*	*ηp* ^2^	*F(p)*	*ηp* ^2^
CBCL internalizing	66.79 (8.01)	65 (7.49)	64.47 (7.92)	61.87 (6.43)	7.279** (0.009)	0.123	0.247 (0.622)	0.005
Withdrawn	67.83 (12.08)	62.75 (10.26)	64.37 (10.76)	60.4 (7)	13.22** (0.001)	0.203	0.201 (0.656)	0.004
Somatic complains	52 (2.28)	51.54 (2.32)	53.40 (4.72)	53.43 (5.13)	0.322 (0.573)	0.006	0.431 (0.514)	0.008
Anxiety/depression	68.79 (8.9)	67.71 (9.17)	65.67 (7.93)	63.40 (7.58)	3.911 (0.053)	0.070	0.488 (0.488)	0.009
Social problems	73 (9.3)	69.67 (9.18)	76.10 (8.23)	72.47 (9.41)	16.29** (<0.001)	0.242	0.004 (0.950)	0.000
Thought problems	67 (7.98)	65.38 (7.79)	63.20 (7.68)	62.03 (7.9)	1.567 (0.216)	0.029	0.042 (0.838)	0.001
CBCL externalizing	66.63 (7.10)	64.79 (6.81)	64.33 (10.43)	61.60 (9.93)	13.178** (0.001)	0.202	0.512 (0.478)	0.010
Inattention	73.08 (8.35)	73.42 (6.68)	73.27 (7.52)	69.73 (7.55)	3.203 (0.079)	0.079	4.676* (0.035)	0.083
Opposition	65.46 (9.76)	63.83 (10.22)	67.03 (11.73)	64 (10.20)	12.128** (0.001)	0.189	1.108 (0.297)	0.021
Conduct problems	63.95 (8.33)	60.87 (7.86)	62.8 (7)	59.23 (6.63)	34.559** (<0.001)	0.399	0.183 (0.671)	0.003
CBCL total	70.17 (3.85)	67.88 (4.46)	67.47 (7.24)	64.50 (7.73)	19.847** (<0.001)	0.276	0.327 (0.570)	0.006
SSIS-P social skills	69.88 (15.58)	73.67 (15.75)	65.63 (16.37)	73.33 (18.32)	21.009** (<0.001)	0.288	2.430 (0.125)	0.045
SSIS-P problem behavior	42.33 (10.32)	39.17 (10.22)	47.80 (16.04)	39.63 (12.57)	40.088** (<0.001)	0.435	7.803** (0.007)	0.130
KIDSCREEN total	67 (8.41)	70.12 (9.32)	66.00 (9.21)	69.00 (9.12)	9.729** (0.003)	0.158	0.144 (0.706)	0.003
Physical well-being	11.33 (3.96)	12.08 (4.47)	12.93 (4.49)	13.70 (3.46)	3.38 (0.072)	0.061	0.000 (0.984)	0.000
Psychological	18.00 (3.20)	17.58 (2.96)	17.57 (4.03)	17.00 (4.67)	0.967 (0.330)	0.018	0.023 (0.881)	0.000
Autonomy and Caregivers	19.42 (4.34)	20.08 (4.58)	18.43 (3.60)	19.23 (4.09)	0.226 (0.142)	0.041	0.018 (0.893)	0.000
Peers and Social	9.67 (2.58)	9.46 (2.06)	8.33 (2.94)	9.63 (2.67)	8.379** (0.006)	0.139	4.389* (0.041)	0.078
Support and School	9.46 (2.90)	9.79 (2.77)	9.40 (2.95)	9.13 (2.73)	0.025 (0.876)	0.000	1.994 (0.164)	0.037
CGI–S–Clinician	4.13 (0.95)	3.11 (0.71)	4.20 (0.76)	3.31 (0.72)	55.877** (<0.001)	0.518	6.54* (0.014)	0.112
PGI–S-parent	4.12 (0.88)	3.13 (0.81)	3.73 (0.87)	2.72 (0.75)	54.023** (<0.001)	0.510	4.938* (0.031)	0.087
BDI–II parents	19.75 (9.72)	19.02 (10.04)	18.63 (9.19)	15.57 (7.37)	6.137* (0.017)	0.106	14.030** (<0.001)	0.212
STAI. state parents	24.29 (11.18)	21.75 (10.03)	26.47 (9.55)	22 (8.56)	40.091** (<0.001)	0.435	3.025 (0.088)	0.055
STAI. trait parents	23.25 (8.87)	21.79 (7.95)	24.23 (9.65)	22.03 (10.87)	12.070** (0.001)	0.188	0.496 (0.484)	0.009

^*^F-values marked with an asterisk indicate significant interaction effects: *p < 0.05; **p < 0.01. CBCL, Child Behavior Checklist; KIDSCREEN-27, Health-Related Quality of Life Questionnaire; CGI-S, Clinical Global Impression-Severity; PGI – S, Parent Global Impression-Severity; SSIS-P, Social Skills Improvement System-Parent; BDI-II, Beck Depression Inventory-II; STAI, State–Trait Anxiety Inventory; ηp^2^, partial eta squared effect size (0.01, small, 0.06, medium, 0.14, large).^*^For significant interactions, paired *t*-tests showed within-group changes (short *n* = 24, long *n* = 31); independent *t*-tests on change scores (df = 54) confirmed between-group differences (see text).

#### 3.3.1 Child behavior checklist (CBCL)

Both groups showed significant improvements over time in internalizing symptoms, withdrawn behavior, social problems, externalizing symptoms, oppositional behavior, and conduct problems (all main effects of time *p* ≤ 0.01; Table 2). A significant time × group interaction was found for inattention (*F*(1, 54) = 4.676, *p* = 0.035, ηp^2^ = 0.083). Follow-up paired-samples *t*-tests indicated a significant reduction in the long-version group from baseline (*M* = 73.27, SD = 7.52) to post-treatment (*M* = 69.73, SD = 7.55; *t*(30) = 2.15, *p* = 0.040), but no significant change in the short-version group (baseline: *M* = 73.08, SD = 8.35; post-treatment: *M* = 73.42, SD = 6.68; *t*(23) = −0.45, *p* = 0.655). An independent-samples *t*-test on change scores confirmed significantly greater improvement in the long-version group (*t*(54) = 2.16, *p* = 0.035).

#### 3.3.2 Social skills and problem behaviors (SSIS-P)

Significant improvements over time were observed for social skills (*F*(1, 54) = 21.009, *p* < 0.001, ηp^2^ = 0.288) and problem behaviors (*F*(1, 54) = 40.088, *p* < 0.001, ηp^2^ = 0.435) in both groups. A significant time × group interaction was found for problem behaviors (*F*(1, 54) = 7.803, *p* = 0.007, ηp^2^ = 0.130). Follow-up paired-samples *t*-tests showed significant reductions in both groups (short-version: baseline *M* = 42.33, SD = 10.32 to post *M* = 39.17, SD = 10.22; *t*(23) = 2.89, *p* = 0.008; long-version: baseline *M* = 47.80, SD = 16.04 to post *M* = 39.63, SD = 12.57; *t*(30) = 4.92, *p* < 0.001), but an independent-samples *t*-test on change scores indicated superior improvement in the long-version group (*t*(54) = 2.80, *p* = 0.007). No significant time × group interaction occurred for social skills (*F*(1, 54) = 2.430, *p* = 0.125).

#### 3.3.3 FASD severity (CGI-S and PGI-I)

Clinician-rated CGI-S severity decreased significantly over time in both groups (*F*(1, 54) = 55.877, *p* < 0.001, ηp^2^ = 0.518), with a significant time × group interaction (*F*(1, 54) = 6.54, *p* = 0.014, ηp^2^ = 0.112). Paired-samples *t*-tests revealed decreases in both groups (short-version: baseline *M* = 4.13, SD = 0.95 to post *M* = 3.11, SD = 0.71; *t*(23) = 5.21, *p* < 0.001; long-version: baseline *M* = 4.20, SD = 0.76 to post *M* = 3.31, SD = 0.72; *t*(30) = 6.89, *p* < 0.001); an independent-samples *t*-test on change scores favored the long-version group (*t*(54) = 2.56, *p* = 0.014). For parent-rated PGI-S, a significant main effect of time emerged (*F*(1, 54) = 54.023, *p* < 0.001, ηp^2^ = 0.510) with a significant interaction (*F*(1, 54) = 4.938, *p* = 0.031, ηp^2^ = 0.087); paired-samples *t*-tests showed reductions (short-version: *t*(23) = 5.67, *p* < 0.001; long-version: *t*(30) = 7.12, *p* < 0.001), and between-group *t*-test on change scores was significant (*t*(54) = 2.22, *p* = 0.031).

#### 3.3.4 Quality of life (KIDSCREEN-27)

Total quality of life improved significantly over time in both groups (*F*(1, 54) = 9.729, *p* = 0.003, ηp^2^ = 0.158), with no significant time × group interaction (*F*(1, 54) = 0.144, *p* = 0.706). A significant interaction was observed for the Peers and Social Support subscale (*F*(1, 54) = 4.389, *p* = 0.041, ηp^2^ = 0.078). Follow-up paired-samples *t*-tests showed improvement in the long-version group (baseline *M* = 8.33, SD = 2.94 to post *M* = 9.63, SD = 2.67; *t*(30) = −3.12, *p* = 0.004) but no meaningful change in the short-version group (baseline *M* = 9.67, SD = 2.58 to post *M* = 9.46, SD = 2.06; *t*(23) = 0.89, *p* = 0.383). An independent-samples *t*-test on change scores confirmed the between-group difference (*t*(54) = 2.10, *p* = 0.041).

#### 3.3.5 Caregivers well-being

State anxiety decreased significantly over time in both groups (*F*(1, 54) = 40.091, *p* < 0.001), with no significant time × group interaction (*F*(1, 54) = 3.025, *p* = 0.088). Trait anxiety also decreased significantly over time (*F*(1, 54) = 12.070, *p* = 0.001), with no interaction (*F*(1, 54) = 0.496, *p* = 0.484). For depressive symptoms (BDI-II), a significant main effect of time was observed (*F*(1, 54) = 6.137, *p* = 0.017), with a significant time × group interaction (*F*(1, 54) = 14.030, *p* < 0.001, ηp^2^ = 0.212). Paired-samples *t*-tests showed a marked reduction in the long-version group (baseline *M* = 18.63, SD = 9.19 to post *M* = 15.57, SD = 7.37; *t*(30) = 2.98, *p* = 0.006), but minimal change in the short-version group (baseline *M* = 19.75, SD = 9.72 to post *M* = 19.02, SD = 10.04; *t*(23) = 0.92, *p* = 0.368). An independent-samples *t*-test on change scores confirmed greater improvement in the long-version group (*t*(54) = 3.75, *p* < 0.001).

## 4 Discussion

This randomized controlled trial advances DAT evidence for FASD by establishing optimal treatment “dose” through the first direct comparison of 8- versus 16-session versions, addressing a critical evidence gap regarding intervention duration effects in canine-assisted interventions where protocols vary widely (6–20 sessions) without head-to-head testing.

Initially, we hypothesized that the longer DAT version would produce superior outcomes compared to the short version. Our findings revealed a nuanced pattern that refined this hypothesis, with treatment duration differentially affecting specific FASD domains. This aligns with our prior research showing DAT efficacy (12- and 16-session versions) for externalizing symptoms and social skills (Vidal et al., 2023; Vidal et al., 2020) but novelly revealing long version superiority in inattention, problem behaviors, FASD severity, social support, and caregiver depression.

These results suggest that more complex domains such as sustained attention and social functioning may require extended intervention to achieve greater gains. In contrast, other externalizing symptoms such as oppositional behavior, conduct problems, and withdrawal appeared responsive to shorter interventions. Importantly, both treatment groups experienced improvements in quality of life, indicating that DAT broadly enhances the clinical profile of children with FASD. The absence of differences in several measures suggests that shorter versions may already provide robust clinical benefits, supporting their use in resource-limited settings unlike longer behavioral therapies requiring sustained dose.

Our secondary hypothesis predicted greater caregiver anxiety and depressive symptom reductions in the longer DAT version. While both versions significantly reduced parental anxiety, only the long-version group demonstrated significantly greater depressive symptom improvement. This dose–response pattern suggests that while shorter interventions effectively address situational anxiety through initial engagement, the chronic burden of caregiver depression -previously linked to FASD behavioral cicles symptoms (Purpura et al., 2021; Tait et al., 2025; Mohamed et al., 2020; Paley et al., 2005) -requires extended treatment duration for meaningful clinical impact. By targeting this key family stressor, the long version functions as a systemic intervention, potentially disrupting the bidirectional caregiver-child stress cycle.

The clinical relevance is underscored by medium-to-large effect sizes (η_p^2^ 0.08–0.21) for key outcomes. Practically, attention/behavior gains may facilitate academic achievement; enhanced social skills can reduce isolation; parental depression relief supports family functioning -all critical for FASD’s lifelong burden. The feasibility and non-invasive nature of DAT support its integration into multimodal treatment plans. The choice between versions can be strategically tailored: shorter versions may offer a scalable solution for immediate symptom relief or resource-limited contexts, while longer interventions may be preferable for targeting complex, ingrained challenges and for providing essential support for parental wellbeing.

These findings advance DAT evidence for FASD by establishing the 16-session version as superior for inattention, problem behaviors, FASD severity, social support, and caregiver depression -building on our prior efficacy RCTs, without prior dose comparisons.

While dose-optimized DAT now offers an empirically supported, engaging intervention for this underserved population, future implementation studies should examine scalability, long-term durability, and cost-effectiveness across diverse FASD services. Optimizing DAT dosing will enhance precision medicine approaches for children with FASD and alleviate associated family burden.

## 5 Limitations and future directions

Despite the valuable insights provided by this study, several limitations warrant consideration. The sample size (*n* = 55) provided adequate power for detecting medium-to-large effects but limited precision for subgroup analyses (e.g., age, FASD subtype) and broader generalizability beyond our Vall d’Hebron FASD clinic population. Non-significant baseline differences in SSIS-P Problem behavior may still have influenced observed change magnitudes.

Concurrent psychopharmacology reflects real-world clinical practice but introduces potential confounds for isolating DAT-specific effects. The absence of longitudinal follow-up limits insights into durability of gains.

Limitations include reliance on pre-enrollment clinical diagnoses rather than study-specific re-assessment, potentially introducing diagnostic variability. However, use of standardized tools (SCID-I/II, K-SADS, WIAT-III) by our multidisciplinary FASD specialist team minimizes this concern. Additional limitations include reliance on parent/clinician ratings -appropriate for this population’s altered self-awareness–though future studies should incorporate educator reports, patient self-reports (when feasible), behavioral observation, and objective biomarkers. Moderator analyses (FASD subtype, ADHD severity, IQ, family factors) remain needed to optimize personalized DAT dosing protocols.

## Data Availability

The raw data supporting the conclusions of this article will be made available by the authors, without undue reservation.
